# The Owens Bill and Its Enemies

**Published:** 1912-01

**Authors:** 


### EDITORIAL DEPARTMENT

The Red Back sends greeting to its readers and wishes them all a prosperous New Year. It is especially grateful for the cordial and long sustained patronage—both subscription and advertising—and is happy.

### THE OWENS BILL AND ITS ENEMIES.

This subject has been discussed in the medical press until it is nearly threadbare. But recent occurrences,—increased activity on the part of the powerful organization known as the "League for Medical Freedom," bring it to the fore again, and render further discussion timely and necessary.

Abuse of this league, denunciation and invectives do no good; it is puerile. It has to be met, and Congress convinced that those heading the opposition to the bill are misrepresenting its aims, scope and purpose, thereby misleading the public and securing thousands of signers to the petition to Congress to defeat the bill. They procure signatures by misstating the case and perverting the meaning of the bill. In all their speeches, publications and lectures they allege that the object of the bill is to create a monopoly of the practice of medicine for the regular (they call it allopathic) profession; and to prevent any person from selecting his medical adviser from any other school of practice. It is truly remarkable that any person should make such statements knowing that they can be easily refuted by simply reading the bill. In it there is not a line or word referring to the practice of medicine, or "school" of medicine. On the contrary, it is distinctly stated (lines 14 and 15, page 3) *"That the Department of Health shall recognize no so-called school or system of medicine."*

That somebody is vitally interested in the defeat of this bill is a fair assumption—when those leading the opposition, men of intelligence and standing resort to willful misrepresentation and to newspaper and magazine publications and to public lectures. They know that the public are not acquainted with the bill and that few if any will take the trouble to investigate it. Under the circumstances such mendacity is criminal. This organization has thousands of dollars to back their crusade, and lecturers are in the field. Newspapers publish their absurd charges without looking at the bill. The medical profession is not more interested in its passage than the humblest citizen. They can not resort to the methods employed by their traducers and opponents of the bill, and hence are almost powerless.

But if the members of every county medical society in Texas would follow the example of the Bexar (Fifth) District Society and the Austin (Seventh) District Society and appeal to the Texas Senators and Representatives by personal letter, it would seem to promise results, for *if they can be induced to look at the bill,* that is all that it is necessary to ask them to do. They will see how utterly false and ridiculous are the so-called grounds of opposition; that there is not a word, line or sentence in the bill that by any kind of distortion can be made to give the faintest semblance of truth to them. The Bexar (Fifth) District Medical Society passed resolutions in which the motives of these parties were impugned (it was true) and addressed their immediate Representative—Congressman Slayden—urging his support. The letter was given to the press, and it called forth a lengthy reply (in the *San Antonio Express*) by a local representative of the "Freedom League" which was remarkable for mendacity and bitterness; it reiterated the absurd charges enumerated above.

The Austin District Medical Society (Seventh) is composed of the physicians of the following (eleven) counties: Bastrop, Burnet, Caldwell, Lee, Llano, San Saba, Travis, Williamson, Mason, Blanco and Hays.

At its annual meeting in Austin December 21, ult., the following resolution was unanimously adopted and a committee (F. E. Daniel, G. P. Smartt and A. Nowlin) was appointed to bring the matter to the attention of the Texas Senators and Congressmen:

*Whereas,* The Owens bill now in Congress providing for the creation of a Department of Public Health under the administration and execution of a Director of Health, is being misrepresented by an organization called the League of Medical Freedom in the daily press and from the rostrum and by circulars and hand bills charging that the aim and object of the bill is to create a monopoly of the practice of medicine in the interest of the regular medical profession, and that no citizen shall have the right to employ any other kind of doctor; and

*Whereas,* These false and absurd charges can be easily disproved by reference to the bill itself, which states (lines 14 and 15, page 3) "That the Department of Health shall recognize no so-called 'school or system of medicine'"; and

*Whereas,* The public are being misled by these misrepresentations and induced to join in the hue and cry of opposition,

*Resolved,* That it is the sense of this body that the Owens bill providing for a Department of Public Health is a wise measure in the interest of the public health, the country's "most valuable asset," and essential to race preservation, and that its passage is demanded by a consideration of these vital objects. We hereby call upon our Congressmen to give it earnest support and urge its passage.

We deplore that the opponents of this measure should so misapprehend its scope and purpose as to publicly charge that it is in the interest of the so-called "regular" profession, and they are hereby challenged to show where in the Owens bill occurs any word or sentence that will justify such charge, or to show anything in the bill that indicates any intention or purpose of placing the administration of such law as is contemplated in the hands of medical men at all. It is a sanitary matter, having for its purpose the application of sanitary principles to the eradication of the causes of disease, and thus, really, making the practice of medicine unnecessary in the sense understood by its opponents.

